# OPTIMIZING PHYSICAL AND MENTAL FUNCTION ACROSS THE AGING CONTINUUM: PCORI’S FUNDING PORTFOLIO AND RESEARCH PRIORITIES

**DOI:** 10.1093/geroni/igae098.1463

**Published:** 2024-12-31

**Authors:** Yewande Akinbami, Tabassum Majid, John Audley, Neeraj Arora

**Affiliations:** Patient-Centered Outcomes Research Institute (PCORI), Washington, District of Columbia, United States; Patient-Centered Outcomes Research Institute (PCORI), Washington, District of Columbia, United States; Patient Centered Outcomes Research Institute (PCORI), Washington, District of Columbia, United States; Patient Centered Outcomes Research Institute, Washington, District of Columbia, United States

## Abstract

In 2021, the National Academy of Medicine identified “Actualizing better health and healthcare for older adults” as one of five vital directions in healthcare. In response, PCORI launched a $50 million, targeted PCORI funding announcement (PFA) on Healthy Aging in 2021 that called for CER to optimize the physical and mental functioning of older adults. This funding announcement coincided with PCORI’s Board identifying 12 focused topic themes, including “Promoting the Health of Older Adults”. This project synthesizes PCORI’s portfolio of funded work aimed at promoting the health of older adults and presents the Healthy Aging continuum, a conceptual framework informed by stakeholders to guide future PCORI research investments. The conceptual framework encompasses four domains: (1) healthy older adults with 1-2 well-managed chronic conditions (2), individuals with sub-optimally managed multiple chronic conditions (3), individuals with significant functional impairment and (4), seriously ill older adults who may need end-of-life care. Caregivers who support older adults as they age throughout the continuum undercut all four of these domains. To date, PCORI has funded 63 CER projects, totaling $323 million, focused on promoting the health of older adults. PCORI’s targeted initiatives have called for CER on palliative care, hearing loss, visual impairment, osteoporotic fractures, delirium, and hypertension. This presentation will provide an overview of PCORI’s portfolio of older adult studies and implications for future research priorities. During the symposium discussion, PCORI will solicit further insights on evidence gaps and implications for future research focused on real-world decisional dilemmas in older adult health.

